# Supplementary material for: Vancomycin-Loaded 3D-Printed Polylactic Acid–Hydroxyapatite Scaffolds for Bone Tissue Engineering
Source: Polymers (Basel). 2023 Oct 28;15(21):4250. doi: 10.3390/polym15214250 (PMC10648244; doi:10.3390/polym15214250)
Supplement: Supplementary file 1 [file polymers-15-04250-s001.zip › polymers-2665389-supplementary.pdf]

PLA SMARTFIL®	Value
Material density	1.24 g/cm^3
Tensile strength (MD)	110 MPa
Tensile modulus (MD)	3.3 GPa
Elongation at break (MD)	160%
Heat deflection temperature	65 °C
Print temperature	200-240 °C

Table S1. Technical data of SMARTFIL® taken from the product data sheet: https://www.smartmaterials3d.com/pla-y-abs-pellets (September 2023). MD means machine direction.

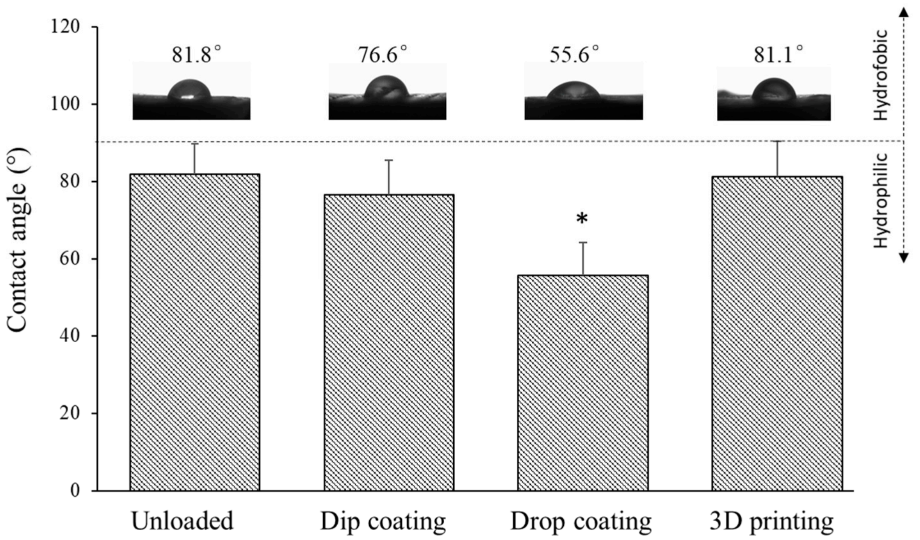

Figure S1. Contact angle measurements at the surface of the 3D printed PLA/HA scaffolds unloaded and after being loaded with vancomycin for the three methodologies. Results are represented as mean ± standard deviation. Statistical significance was determined at * $p \leq 0.05$.
